# Cytomegalovirus infection exacerbates autoimmune mediated neuroinflammation

**DOI:** 10.1038/s41598-017-00645-3

**Published:** 2017-04-06

**Authors:** Marjan Vanheusden, Bieke Broux, Suzanne P. M. Welten, Liesbet M. Peeters, Eleni Panagioti, Bart Van Wijmeersch, Veerle Somers, Piet Stinissen, Ramon Arens, Niels Hellings

**Affiliations:** 1 Hasselt University, Biomedical Research Institute and Transnationale Universiteit Limburg, School of Life Sciences, Diepenbeek, Belgium; 2 Leiden University Medical Centre, Department of Immunohematology and Blood Transfusion, Leiden, The Netherlands; 3 Rehabilitation and Multiple Sclerosis Centre, Overpelt, Belgium

## Abstract

Cytomegalovirus (CMV) is a latent virus which causes chronic activation of the immune system. Here, we demonstrate that cytotoxic and pro-inflammatory CD4^+^CD28^null^ T cells are only present in CMV seropositive donors and that CMV-specific Immunoglobulin (Ig) G titers correlate with the percentage of these cells. *In vitro* stimulation of peripheral blood mononuclear cells with CMVpp65 peptide resulted in the expansion of pre-existing CD4^+^CD28^null^ T cells. *In vivo*, we observed *de novo* formation, as well as expansion of CD4^+^CD28^null^ T cells in two different chronic inflammation models, namely the murine CMV (MCMV) model and the experimental autoimmune encephalomyelitis (EAE) model for multiple sclerosis (MS). In EAE, the percentage of peripheral CD4^+^CD28^null^ T cells correlated with disease severity. Pre-exposure to MCMV further aggravated EAE symptoms, which was paralleled by peripheral expansion of CD4^+^CD28^null^ T cells, increased splenocyte MOG reactivity and higher levels of spinal cord demyelination. Cytotoxic CD4^+^ T cells were identified in demyelinated spinal cord regions, suggesting that peripherally expanded CD4^+^CD28^null^ T cells migrate towards the central nervous system to inflict damage. Taken together, we demonstrate that CMV drives the expansion of CD4^+^CD28^null^ T cells, thereby boosting the activation of disease-specific CD4^+^ T cells and aggravating autoimmune mediated inflammation and demyelination.

## Introduction

Multiple sclerosis (MS) is a disabling autoimmune disease of the central nervous system (CNS). Activated autoreactive immune cells infiltrate the brain and spinal cord leading to chronic inflammation, demyelination and ultimately axonal loss^1^. Although the exact trigger for this activation has not been elucidated yet, a genetic predisposition in combination with environmental factors seems essential to develop MS^2^. Worldwide, about 2.5 million people are affected, mostly young adults (20–40 y) and females (3:1 ratio), although the disease progression in men can be more severe^3^.

Naive T cells express CD28 on their cell surface, but due to repeated antigenic stimulation CD28 expression can be lost^4–6^. CD4^+^CD28^null^ memory T cells arise during chronic activation of the immune system, in a subset of healthy controls (HC) and patients with MS. These cells have a restricted T cell receptor (TCR) diversity (oligoclonal), are costimulation independent, more resistant to apoptosis, and less susceptible to suppression by regulatory T cells (Tregs)^7–12^. Relevant features suggesting their contribution to autoimmune mediated CNS damage in MS include their autoreactive nature; their target tissue infiltration, via e.g. the fractalkine gradient; and their cytotoxic capacities, namely the expression of natural killer (NK) cell receptors and the production of perforin and granzymes^11, 13, 14^.

So far, the trigger for the selective expansion of CD4^+^CD28^null^ T cells and their contribution to MS disease pathology is poorly investigated. There is mounting evidence that CD4^+^CD28^null^ T cell expansion occurs after infection with cytomegalovirus (CMV)^9, 15–17^. CMV is a member of the β-herpesvirus family that establishes lifelong latent infections in ≥70% of the human population^18^. CMV commits a large portion of its genome to evade recognition and activation of the immune system: e.g. reduction of antigen presentation by interfering with the expression of MHC/HLA molecules, downmodulation of costimulatory molecules, and evasion of NK cell control^16, 19–21^. However, as a result of cross-priming of CMV antigens, CMV-specific T cell responses develop. Moreover, due to the persistent nature of CMV, substantial accumulation of CMV-specific memory T cells (on average 10% of the total memory T cell compartment) can occur^18, 22–24^, albeit with varying degrees, which may be caused by differences in infectious dose^25^. As a consequence of this large percentage of CMV-specific T cells, immune surveillance could become less effective over time, thereby compromising normal immunity^18, 26^. Indeed, CMV seropositivity has been correlated with a worse MS disease course, although disease limiting effects have also been stated (Reviewed in ref. 16). The most important finding indicating a disease promoting role is the enrichment of CMV-specific antibodies in MS^27^. When these antibodies were present in MS patients, this was correlated to a decreased time to relapse, an increase in the number of relapses and enhanced brain atrophy^28–30^. In contrast, another study concluded that the presence of CMV-specific antibodies was associated with a better clinical outcome, an increased age of disease onset and decreased brain atrophy^31^. A recent meta-analysis on 1341 MS patients and 2042 healthy controls did not yield a conclusive result on the relationship between CMV infection and the occurrence of MS^32^.

In this study we investigated whether CMV by itself is able to trigger the expansion of CD4^+^CD28^null^ T cells and aggravate MS disease, using a combination of human data and *in vivo* animal model systems.

## Results

### CMV expands CD4^+^CD28^null^ T cells via repeated antigenic stimulation

**Figure 1:**
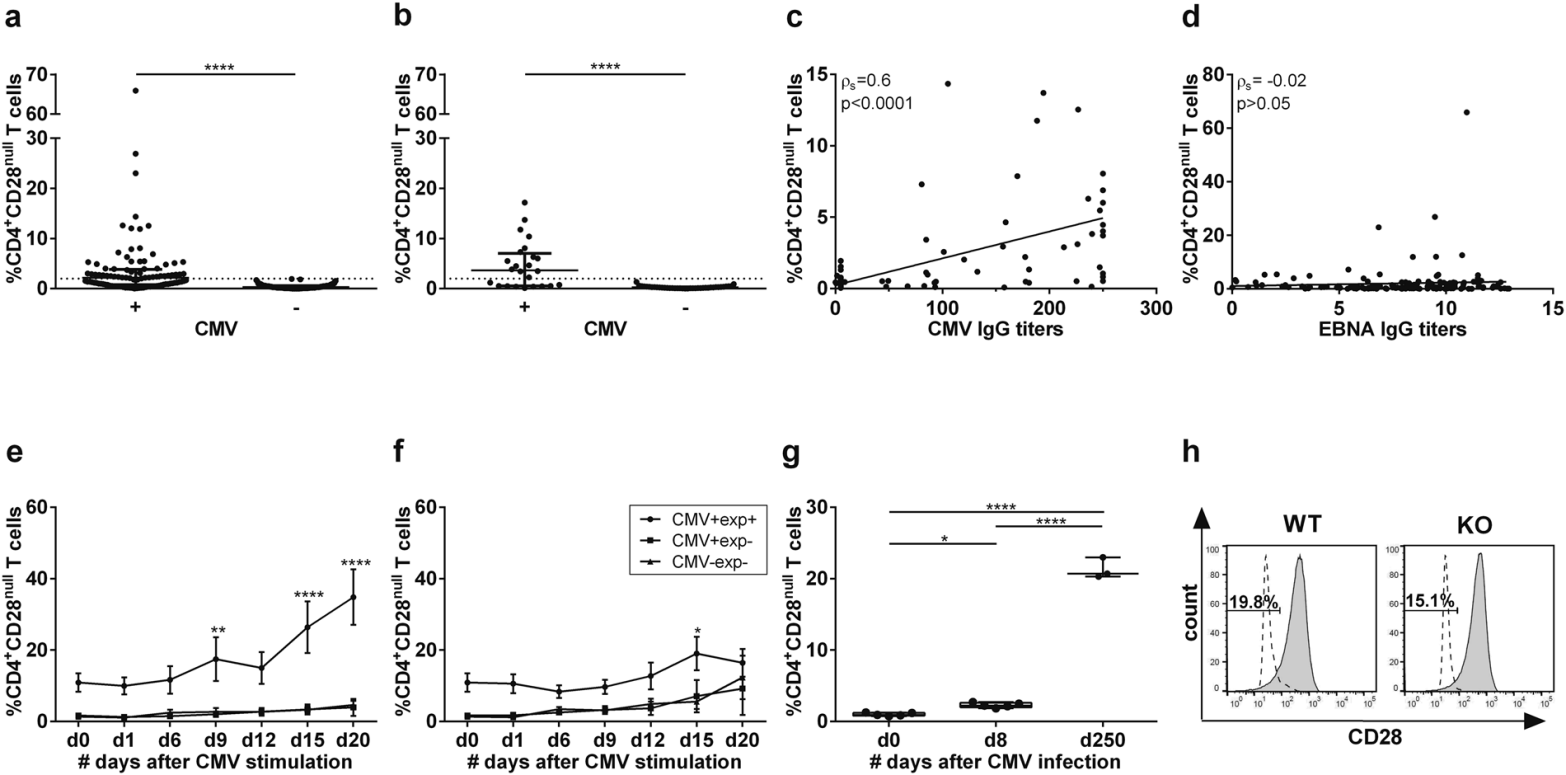
CMV infection expands CD4^+^CD28^null^ T cells. Flow cytometry was performed to determine the percentage of CD4^+^CD28^null^ T cells. CMV and EBV status and immunoglobulin titers were determined via ELFA. CD4^+^CD28^null^ T cells in (**a**) CMV seropositive (n = 100) compared to seronegative (n = 127) MS patients and (**b**) CMV seropositive (n = 24) versus seronegative healthy controls (n = 39). (**c**) Correlation of CMV IgG levels with the percentage of CD4^+^CD28^null^ T cells in MS patients and HC (n = 140). (**d**) Correlation of EBV EBNA IgG titers in 155 MS patients. (**e**) Repeated stimulation of PBMCs from HC (n = 12) and MS patients (n = 8) with CMV pp65 (**e**) or IL-2 (**f**) *in vitro*, after which the number of CD4^+^CD28^null^ T cells was determined at different time points. (**g**) Flow cytometry of splenocytes of MCMV infected mice on day 0, day 8 and day 250 post infection (n = 5/time point). (**h**) Splenocytes from MCMV-infected WT (n = 5) and CD80/86^−/−^ (n = 4) mice were analysed for the percentage of CD4^+^CD28^null^ T cells at day 250 post infection. *p < 0.05, **p < 0.01, ****p < 0.0001.

To determine whether CMV infection is linked to expansion of CD4^+^CD28^null^ T cells (>2% of CD4^+^ T cells), an association study between CMV serology and the percentage of CD4^+^CD28^null^ T cells was performed. In our cohort, the percentage of CD4^+^CD28^null^ T cells is significantly higher in CMV seropositive (CMV+) donors compared to CMV seronegative (CMV−) donors (p < 0.0001, Fig. 1a and b), with no differences between MS and HC, which is in line with other studies^9^. Furthermore, CMV-specific IgG titers positively correlate with the percentage of CD4^+^CD28^null^ T cells (ρ_s_ = 0.6, p < 0.0001, Fig. 1c). To test whether this correlation is CMV specific, we examined the serology of EBV, another chronic and latent virus which has been implicated in MS^33^. No significant correlation was found between the percentage of CD4^+^CD28^null^ T cells and EBNA IgG titers (Fig. 1d). Furthermore, EBV IgG levels did not differ between donors with versus without CD4^+^CD28^null^ T cell expansion (respectively: 9 ± 4 vs 8 ± 4, p > 0.05). In contrast, donors with CD4^+^CD28^null^ T cell expansion have significantly higher CMV IgG titers compared to donors without expansion (respectively: 219 ± 92.8 vs 5 ± 0, p < 0.0001).

Since CD4^+^CD28^null^ T cell expansion only occurred in CMV infected individuals and correlated with the level of CMV-specific antibody titers, we investigated whether CMV infection can drive expansion of CD4^+^CD28^null^ T cells, using *in vitro* and *in vivo* models. Since there is no significant difference in the percentage of CD4+CD28^null^ T cells between HC and MS patients, we did not discriminate between both populations in the following experiment. To mimic chronic TCR triggering by CMV, PBMCs from MS patients and HC, who were either CMV+ or CMV− and exhibited CD4^+^CD28^null^ T cell expansion (exp+) or not (exp−), were repeatedly stimulated with a CMV peptide (CMVpp65) *in vitro*. The percentage of CD4^+^CD28^null^ T cells significantly increased over time in CMV+ exp+ donors, as opposed to CMV+ exp− and CMV−exp− donors (Fig. 1e). IL-2 by itself did not induce expansion of CD4+CD28^null^ T cells (Fig. 1f). Repetitive CMV peptide stimulation *in vitro* did not induce the generation of CD4^+^CD28^null^ T cells in exp− donors over the duration of the experiment (20 days). To investigate the long term effect of CMV infection on formation and expansion of CD4^+^CD28^null^ T cells, we used the *in vivo* MCMV mouse model, the most widely used and relevant model for human CMV infection^25^. MCMV infected mice showed a significant increase of CD4^+^CD28^null^ T cells in the spleen over time, with a 2-fold increase at day 8 (p < 0.05) and 20-fold increase at day 250 post-infection compared to non-infected mice (d0, p < 0.0001, Fig. 1g). In non-infected mice, the CD4^+^CD28^null^ T cell levels were below the threshold for expansion (1 ± 0.2%), indicating that CMV infection induces loss of CD28 in CD4^+^ T cells *in vivo*. In summary, repeated *in vitro* stimulation with CMV peptide expands pre-existing CD4^+^CD28^null^ T cells, whereas *in vivo* CMV infection induces CD28 loss in CD4^+^ T cells and drives expansion of CD4^+^CD28^null^ T cells.

To determine whether CMV induces the loss of CD28 on CD4^**+**^ T cells via repeated antigenic triggering or via interaction with its ligands CD80 and CD86, we infected CD80/86^−/−^ mice with MCMV. MCMV infection induced the expansion of CD4^+^CD28^null^ T cells to a similar extent in CD80/86^−/−^ mice and WT mice (Fig. 1h), indicating that the loss of CD28 is not caused by binding with their ligands CD80 and CD86. These findings further strengthen our notion that CD28 loss is caused by repeated antigenic triggering via the TCR.

### CD4^+^CD28^null^ T cells are increased in EAE mice and correlate with disease severity

**Figure 2:**
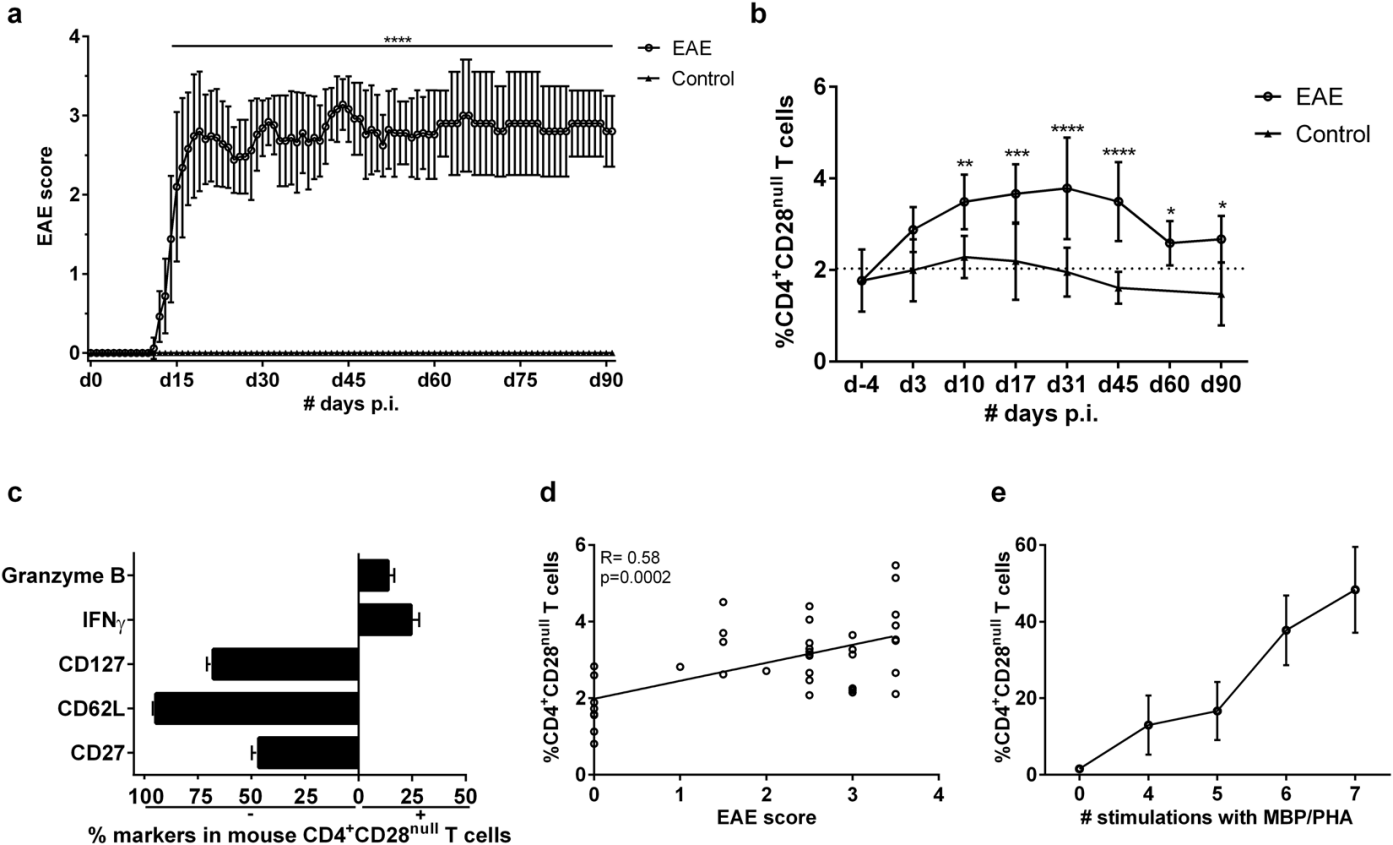
CD4^+^CD28^null^ T cells are increased in EAE mice, as a result of auto-antigenic stimulation. (**a**) The phenotype of blood-derived mouse CD4^+^CD28^null^ T cells was measured via flow cytometry. (**b**) After induction, EAE mice (n = 15) and CFA control mice (n = 10) were scored for maximum 90 days according to their disability. (**c**) Blood was collected at different time point**s**, to determine the number of CD4^+^CD28^null^ T cells via flow cytometry. (**d**) Correlation between CD4^+^CD28^null^ T cells and EAE score. (**e**) Historical human MBP specific T cell clones repeatedly stimulated with MBP/PHA (n = 8) were thawed and analysed for the number of CD4^+^CD28^null^ T cells via flow cytometry. **p < 0.01, ***p < 0.001, ****p < 0.0001.

CD4^+^CD28^null^ T cells are cytotoxic, accumulate in MS lesions and at least a subpopulation is autoreactive in nature^14^. To test the hypothesis that CD4^+^CD28^null^ T cells are associated with the severity of neuroinflammation, an EAE experiment was performed. Follow-up time (Fig. 2a) was extended compared to the standard protocol (30 day p.i.), to test whether CD4^+^CD28^null^ T cells expand during acute and chronic stages of EAE (Fig. 2b). While limited numbers of CD4^+^CD28^null^ T cells were found in CFA control mice, a significant increase above the 2% threshold for expansion was only found in the EAE mice (EAE: 3 ± 0.7%, p = 0.004 and control: 1.8 ± 0.3%, p > 0.05, Fig. 2b).

From previous studies, it is known that human CD4^+^CD28^null^ T cells produce IFNγ and granzyme B, and that they show low expression of CD62L, CD127 and CD27^9, 14, 34–36^. To determine whether mouse CD4^+^CD28^null^ T cells have a similar phenotype, we analysed these cells, which were present in the peripheral blood of EAE mice. We found that they indeed phenotypically resemble their human counterparts as evidenced by a low expression of CD62L, CD127 and CD27, and production of IFNγ and granzyme B (Fig. 2c), identifying them as proinflammatory and cytotoxic effector memory T cells.

Furthermore, the percentage of CD4^+^CD28^null^ T cells positively correlated with the EAE disease score (ρ_s_ = 0.6, p = 0.0002, Fig. 2d). Long-term follow-up indicated that there was no further expansion of CD4^+^CD28^null^ T cells in the chronic phase of EAE (after d30).

The increase in CD4^+^CD28^null^ T cells in EAE mice could result from repeated auto-antigenic stimulation. To test this hypothesis, human MBP-specific T cell clones, generated and sustained *in vitro* by stimulation rounds with MBP or PHA, were analysed for the presence of CD4^+^CD28^null^ T cells (Fig. 2e). The number of CD4^+^CD28^null^ T cells increased after each successive round of stimulation. Thus, repeated MBP stimulation leads to the expansion of CD4^+^CD28^null^ T cells *in vitro*, indicating that the expansion of CD4^+^CD28^null^ T cells in MS patients may result from chronic auto-antigenic stimulation *in vivo*. Of note, *in vitro* stimulation with tetanus toxoid also induced expansion of CD4^+^CD28^null^ T cells (Supplementary Figure S2), indicating that the expansion is not antigen specific, but rather due to the chronicity of the antigen exposure.

### CMV infection exacerbates clinical symptoms of EAE

**Figure 3:**
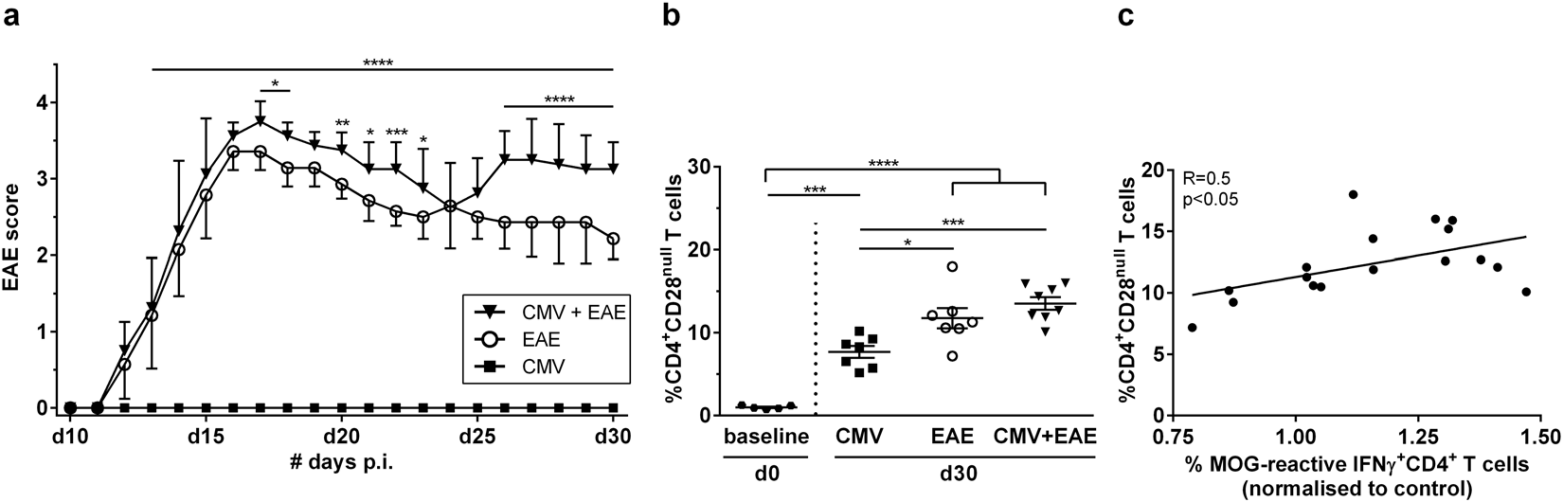
MCMV infected mice with EAE have a worse disease course. Mice were infected with MCMV and after 8 days EAE was induced. (**a**) Daily scoring of the CMV, EAE and MCMV infected EAE groups (n = 8/group). (**b**) Splenocytes were isolated at day 0 (baseline) and day 30 and CD4^+^CD28^null^ T cells were measured via flow cytometry. (**c**) After stimulation of splenocytes with a MOG_35–55_ peptide, the IFNγ producing CD4^+^ T cells were measured via flow cytometry and normalized to the non-peptide control. The MOG response significantly correlated with the percentage of CD4^+^CD28^null^ T cells in the spleen. *p < 0.05, **p < 0.01, ***p < 0.001, ****p < 0.0001.

Our results indicate that CD4^+^CD28^null^ T cells expand after repeated immune activation, either as a result of CMV infection or after the induction of autoimmunity. Here, we investigated whether CMV infection and subsequent expansion of CD4^+^CD28^null^ T cells correlate with a worse EAE outcome. The interplay between these different factors was investigated by infecting mice with MCMV and subsequently inducing EAE 8 days later. The EAE disease score of mice that were pre-exposed to MCMV was significantly higher compared to the EAE control group (mean cumulative score: 56 ± 4 vs 47 ± 3, p < 0.01; mean maximal score: 3.8 ± 0.26 vs 3.5 ± 0, p < 0.02; mean end score: 3.1 ± 0.35 vs 2.2 ± 0.27 p = 0.002). Furthermore, the MCMV group experienced a relapse between day 26 and day 30 after immunization, whereas EAE control mice did not (Fig. 3a). The percentage of CD4^+^CD28^null^ T cells in the spleen increased at least eight-fold in each group (CMV: 8 ± 2%, p < 0.001, EAE: 12 ± 3%, p < 0.0001 and CMV+ EAE: 14 ± 2%, p < 0.0001) compared to baseline (1 ± 0.2%) (Fig. 3b). These results provide further evidence that both CMV infection and EAE induction lead to the expansion of CD4^+^CD28^null^ T cells and that prior CMV infection aggravates EAE symptoms.

Since we showed that CMV exacerbates EAE disease, we asked whether this is due to increased autoimmune reactivity. To answer this question, CD4^+^ T cell reactivity to MOG peptide was measured in the spleen. The MCMV infected EAE group displayed enhanced MOG-specific CD4^+^ T cell reactivity compared to the control groups (EAE: p < 0.004, CMV: p < 0.002). Furthermore, this MOG response correlated to the percentage of CD4^+^CD28^null^ T cells in the spleen of these mice (Fig. 3c). Also, we detected splenic CMV-specific CD4^+^ T cell reactivity in the MCMV infected groups, however they were not increased by EAE induction (data not shown). Viral load measured in the salivary glands at the end of the experiment indicate that the virus was still present in high amounts in both the MCMV and the MCMV infected EAE groups (data not shown). These data indicate that CMV infection increases the percentage of MOG-specific CD4^+^ T cells, thereby increasing autoimmune mediated neuroinflammation, and that CD4^+^CD28^null^ T cells take part in this overall MOG response.

### CMV infection increases demyelination in EAE

**Figure 4:**
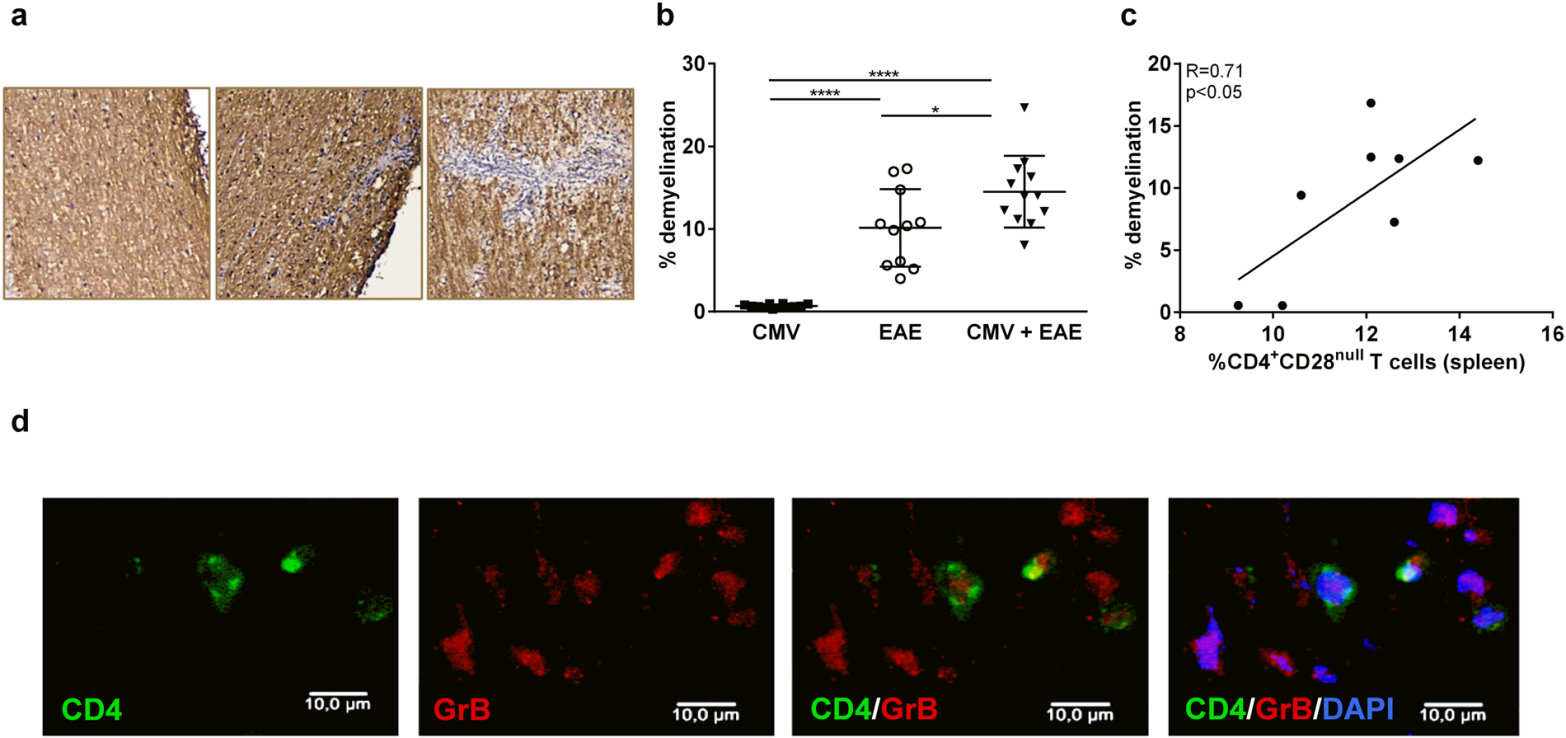
CMV infection increases EAE lesion size. (**a**) Representative staining of MBP in the CMV control group (left), the EAE control group (middle) and the CMV infected EAE animals (right). (**b**) The amount of demyelination within the CMV and EAE control groups and the CMV infected EAE group, calculated via dividing the demyelinated area (=loss of MBP) in the white matter of the spinal cord, over the total white matter area for each section. (**c**) Correlation between the amount of demyelination for each animal and the percentage of peripheral CD4^+^CD28^null^ T cells. (**d**) Single and double staining of CD4 and Granzyme B, which points to the presence of CD4^+^CD28^null^ T cells, in the spinal cord of EAE mice. *p < 0.05, ****p < 0.0001.

In MS patients, CD4^+^CD28^null^ T cells accumulate in brain lesions and are in close contact with neural cells^14^. Since CMV infection leads to a worse EAE disease course, we next questioned whether demyelination of the spinal cord, the predominant location of lesions in this model, is also increased in these animals. No demyelination was found in the spinal cord of MCMV infected mice (Fig. 4a and b). MCMV infected EAE animals exhibited enhanced demyelination compared to the EAE control group (Fig. 4b), indicating that CMV infection accelerates autoimmune-mediated CNS damage. Furthermore, the extent of demyelination is strongly correlated with the percentage of spleen-derived CD4^+^CD28^null^ T cells (R = 0.71, p < 0.05, Fig. 4c). We further identified the presence of CD4^+^GranzymeB^+^ T cells in the spinal cord (Fig. 4d), suggesting that CD4^+^CD28^null^ T cells, which are granzyme B^+^, are present in the spinal cord and possibly contribute to CNS damage.

## Discussion

Here, we demonstrate that CD4^+^CD28^null^ T cells expand during EAE and positively correlate with disease severity. In addition, we show that CMV by itself is able to enhance activation of disease-specific CD4^+^ T cells, trigger the expansion of CD4^+^CD28^null^ T cells and worsen EAE. Overall, our findings support a detrimental role for CMV in autoimmune neuroinflammation.

Our group, together with others have shown that CD4^+^CD28^null^ T cells are associated with the pathogenesis of chronic inflammatory disorders^15, 37, 38^. In MS, a direct link with disease severity has not been demonstrated so far. However, indirect evidence, such as their target tissue infiltrating capacity and cytotoxic activity towards oligodendrocytes, certainly alludes to this hypothesis^14, 35^. In this study, we made use of the widely documented mouse model for MS, EAE. Although this model is certainly not fully equivalent to the human situation, it does recapitulate the inflammatory response that arises in patients with MS, which is the focal point of our study^39^. Here, we demonstrate that peripheral CD4^+^CD28^null^ T cells are increased in EAE animals and that the percentage of CD4^+^CD28^null^ T cells is strongly correlated with the amount of demyelination and disease severity. Mouse-derived CD4^+^CD28^null^ T cells displayed an effector memory (CD62L^low^CD127^low^CD27^low^IFNγ^+^) and cytotoxic (granzyme B^+^) phenotype, indicating that they are similar to their human counterparts^9, 14, 34–36^. Our findings are in line with evidence found in collagen-induced arthritis (CIA), the animal model for RA, where an increase in the number of CD4^+^CD28^−^NKG2D^+^ T cells was observed after immunization^40^. The increase in peripheral CD4^+^CD28^null^ T cells in EAE mice could be attributed to repeated auto-antigenic stimulation caused by chronic autoimmune inflammation. Indeed, as evidenced by our *in vitro* data, repeated MBP stimulation of MBP-specific T cell clones leads to CD4^+^CD28^null^ T cell expansion. *In vivo*, we found a direct correlation between the percentage of CD4^+^CD28^null^ T cells and the anti-MOG response level in the spleen of EAE mice. Together, these findings confirm the autoreactive nature of CD4^**+**^CD28^null^ T cells^11^. After 30 days p.i., there was no further expansion of CD4^+^CD28^null^ T cells in the blood of EAE mice. Instead, starting from day 60, the memory pool maintained a steady state. This is as expected with regards to the homeostasis of the memory pool: expansion is followed by contraction and ultimately maintenance of the remaining memory T cell pool^41^.

In contrast to EAE mice, not all MS patients have CD4^+^CD28^null^ T cell expansion. Therefore, in humans additional components could be important in the generation of CD4^+^CD28^null^ T cells. Potential triggers include: 1) chronic inflammation^42^; and 2) viral infections^7^, of which CMV, as a persistent virus, is a promising candidate. Our data demonstrate that repetitive *in vitro* CMV peptide stimulation of human PBMCs expands pre-existing CD4^+^CD28^null^ T cells. IL-2, which enhances T-cell proliferation and differentiation, does not lead to the expansion of CD4^+^CD28^null^ T cells. EBV, another chronic and latent virus implicated in MS, is not associated with CD4^+^CD28^null^ T cell expansion. These findings further support the hypothesis that CD4^+^CD28^null^ T cells arise after CMV infection, which corresponds with previous reports by other groups^9, 17^. Of note, we did not measure proliferation; therefore the increase in CD4^+^CD28^null^ T cells after CMV stimulation could be due to survival rather than proliferation. However, van Leeuwen *et al*. indicated that CD4^+^CD28^null^ T cells proliferate after addition of CMV antigens, suggesting the latter is true^17^. *In vivo*, CMV infection leads to continuous activation, enabling us to study chronic repeated antigenic challenge. Although human CMV and MCMV are different viruses, the MCMV mouse model is widely used and is the most relevant mouse model which mimics human CMV infection^25^. MCMV virus in the salivary gland is thought to be important for spreading the virus from mouse to mouse. Whereas in all organs the virus is latent in less than a few weeks, in the salivary glands the virus replicates for months^43^. Thus the amount of virus in the salivary gland is not influencing the titers in other organs, such as spleen and lymph nodes, but is instead set by the initial infection dose, and the local and pre-existing immunity conditions. Using this model, we clearly show formation and expansion of CD4^+^CD28^null^ T cells in all MCMV infected animals over time. These findings are in line with those of other groups^7^. Since CMV is unable to infect T cells, CMV cannot directly reduce CD28 expression on T cells, but rather exerts its effects due to its persistent nature. In this study, we show that the loss of CD28 is caused by continued antigenic triggering and not by binding with their ligands CD80 and CD86, since the number of CD4^+^CD28^null^ T cells did not differ between MCMV-infected CD80/86^−/−^ mice and WT. Furthermore, studies in mice and humans have indicated that the number and phenotypes of CMV-specific T cells correlate with viral load^25, 44, 45^; higher viral loads drive higher expansions, establishing the antigen-driven aspect of the response. In this study, we used a relatively high dose of MCMV leading to a higher amount of antigen-specific T cells, including CD4^+^CD28^null^ T cells. In the human population, the dose of CMV is not evenly distributed, leading to variability in the number of antigen specific T cells between individuals. This heterogeneity explains the difference in the percentage of CD4^+^CD28^null^ T cells among CMV seropositive donors. In this respect, it is of interest to note that the CMV Ig titers correlate with a higher percentage of CD4^+^CD28^null^ T cells. This implies that individuals with a higher CMV exposure may develop more CD4^+^CD28^null^ T cells and associated disease. Also, the proportion of CMV-seropositive individuals increases with age^46^, as does the percentage of CD4^+^CD28^null^ T cells^47^.

In MS patients, CMV seropositivity and high IgG titers are correlated with increased percentages of CD4^+^CD28^null^ T cells. This link between CMV and CD4^+^CD28^null^ T cell expansion was previously also reported for RA, ankylosing spondylitis and cardiovascular diseases^17, 38, 48–52^, indicating that CMV infection and CD28^null^ T cell expansion form a common pathogenic background in these diseases^9^. The logical next step is to confirm the possible link between CMV, CD4^+^CD28^null^ T cells and autoimmunity. Here, we demonstrate that CD4^+^CD28^null^ T cells are increased in MCMV, EAE and MCMV+ EAE mice after 30 days p.i. MCMV infected EAE animals had a higher disability score and experienced a relapse, compared to the EAE control mice. Furthermore, MCMV infection increased demyelination in EAE mice, which correlated with higher CD4^+^CD28^null^ T cell percentages in the periphery. Since we found CD4^+^GranzymeB^+^ T cells in the spinal cord of EAE and MCMV infected EAE mice, this suggests that CD4^+^CD28^null^ T cells accumulate in the CNS to inflict damage in line with our previous observations in post-mortem MS brain material^14^. Thus, CMV infection exacerbates EAE disease course and does this by boosting the autoimmune response, as indicated by an increased MOG response. Indeed, T cell expansion preferentially occurred in MOG specific T cells, since the overall T cell responsiveness (no peptide control) in the spleen was comparable between all groups (data not shown). This is in accordance with others, where EAE induction combined with viral infection (γ-herpes virus, Semlike Forest virus or Sindbis virus) accelerated or exacerbated disease as a result of enhanced immune cell infiltration and polarization of the adaptive immune response^53–55^. Furthermore, MCMV infection rendered EAE-resistant BALB/c mice susceptible for EAE induction^56^. In another murine model of MS, namely Theiler’s murine encephalitis virus (TMEV) model, opposite findings were demonstrated; CMV infection attenuated TMEV disease course^57^. However, the immune response in TMEV is largely CD8 mediated, whereas in EAE and MS CD4^**+**^ T cells are the main players^58^. We believe that the EAE model better represents what is going on in MS, namely a primary autoimmune mediated attack of the CNS, in contrast to the TMEV model, where primary viral induced neurotoxicity induces secondary autoimmunity.

An important question still remains to be answered: is the disease exacerbating effect and enhanced demyelination directly caused by CMV infection itself or attributable to the increased expansion of CD4^+^CD28^null^ T cells? While technically challenging, an adoptive transfer study is needed to indisputably prove a direct cause-and-effect relationship of CD4^**+**^CD28^null^ T cells and disease severity. CMV was previously reported to be present in the CNS, where it could damage local cells and tissues directly^33^. The ensuing cell death could then enhance autoimmunity as a result of the release and spreading of self-epitopes from degenerating tissue^59^. However, since demyelination was not present in animals only infected with CMV, it is unlikely that CMV by itself leads to CNS damage as proposed by the epitope spreading hypothesis. On the other hand, reactivation of CMV during ongoing MS could trigger the activation of autoreactive T cells (molecular mimicry) thereby enhancing subsequent demyelination. Of note, CMV-specific T cells were previously identified in MS lesions^60^. Evidence for cross reactivity between a CMV antigen (UL86_981–1003_) and the myelin oligodendrocyte glycoprotein epitope (MOG_35–55_) has been found in rats and non-human primates^61, 62^. However, our data show that CMV infection alone did not mount a significant MOG response in the spleen, which would have been the case if molecular mimicry was involved. Another possible way by which CMV could directly contribute to autoimmunity is through bystander activation, where the immune response against CMV leads to robust inflammation, triggering the non-specific activation of autoreactive T cells^63^. We postulate that these bystander activated autoreactive T cells are mainly responsible for exacerbating EAE disease severity.

In summary, CMV infection and EAE induction lead to the expansion of CD4^+^CD28^null^ T cells. Both CMV infection and CD4^+^CD28^null^ T cells aggravate autoimmune mediated CNS inflammation, since EAE disease severity, measured by EAE score and the extent of neuroinflammation and demyelination, correlated with increasing amounts of CD4^+^CD28^null^ T cells and the presence of a CMV infection. Overall, CMV infection drives the expansion of CD4^+^CD28^null^ T cells, thereby amplifying the activation of disease-specific CD4^+^ T cells, and exacerbating EAE disease. Future studies will address whether this is also the case in MS patients. However, CMV vaccination to prevent the formation of CD4^+^CD28^null^ T cells and the adverse effects of the infection itself, could be beneficial for people at risk of developing MS.

## Methods

### Study subjects

#### Human

Peripheral blood samples (Li-Heparin coated tubes) were collected from 63 healthy controls (HC) and 227 MS patients in collaboration with the University Biobank Limburg (UBiLim). CMV and Epstein-Barr virus (EBV) status and titers (CMV IgG and EBV EBNA IgG) were determined in serum samples via Vidas ELFA (bioMérieux, Marcy l’Etoile, France) and Architect immunoassay (Abbott, Illinois, USA). Clinical data are presented in Table 1; there were no significant differences between CMV positive or negative donors, neither in MS patients nor in healthy controls.

**Table 1:** Study subjects for CD4^+^CD28^null^ T cell analysis.

	MS patients		Healthy controls	
	CMV+	CMV−	CMV+	CMV−
Number	100	127	24	39
Age (y)	47 ± 13	44 ± 14	32 ± 9	32 ± 10
Male/Female (ratio)	26/74 (0.35)	35/92 (0.38)	7/17 (0.41)	14/25 (0.56)
EBV serostatus (−/border/+)	0/2/64	2/0/87	NA	
Disease duration range	1 mo–40 y	0 mo–37 y	NA	
EDSS range	0–7	0–7.5	NA	
Disease type			NA	
CIS	5	6		
RR-MS	63	78		
CP-MS	32	43		
Treatment^#^			NA	
No treatment	46	56		
IFNβ	25	47		
Glatiramer acetate	15	10		
Natalizumab	9	6		
Alemtuzumab	2	4		
Teriflunomide	/	3		
Dimethyl fumarate	2	/		
Methotrexate	1	1		

^#^Within 3 months before blood collection.

MS, multiple sclerosis; EDSS, expanded disability status scale; CIS, clinically isolated syndrome; RR, relapsing remitting; CP, chronic progressive; IFNβ, interferon beta; CMV, cytomegalovirus; NA, not applicable.

#### Mice

Female C57BL/6 mice were purchased from Harlan (Horst, the Netherlands). CD80/86^−/−^ mice^64^ were bred in LUMC to the C57BL/6 background.

### EAE induction

10 week old C57BL/6J mice were immunized subcutaneously with myelin oligodendrocyte glycoprotein 35–55 peptide (MOG_35–55_) emulsified in complete Freund’s adjuvant (CFA) containing Mycobacterium tuberculosis according to manufacturer’s guidelines (Hooke Laboratories, Lawrence, USA). Directly after immunization and 24 h later, mice were intraperitoneally injected with pertussis toxin. Mice were weighed and evaluated daily for neurological signs of disease using a standard 5-point scale; 0: no symptoms; 1: limp tail; 2: hind limp weakness; 3: complete hind limp paralysis; 4: complete hind limp paralysis and partial front leg paralysis; 5: moribund.

### MCMV infection

MCMV-Smith was obtained from the American Type Culture Collection (ATCC, Manassas, VA) and stocks were prepared from the salivary glands of infected BALB/c mice. C57BL/6J WT and CD80/86^−/−^ mice were infected i.p. with 5 × 10^4^ PFU. All mice were maintained under specific pathogen free conditions.

### Flow cytometry

#### Human

All donors included in this study were analysed for the percentage of CD4^+^CD28^null^ T cells. This was done by isolating peripheral blood mononuclear cells (PBMCs) from whole blood by density gradient centrifugation (Cedarlane lympholyte, Sheffield, UK). Cells were double stained with anti-human CD4 FITC and CD28 PE (both BD Biosciences, Franklin Lakes, NJ). The gating strategy consists of a lymphocyte gate using the forward and side scatter signal, after which CD4^+^ cells were gated and subsequently CD28 expression was monitored within this gate (Supplementary Figure S1a). Cells were acquired using a FACSAria II cytometer, and data were analysed using BD FACSDiva software. Significant expansion of CD4^+^CD28^null^ T cells was arbitrary defined as a percentage ≥2% of the total CD4^+^ T cell population, as this was the minimal percentage of cells that allowed discrimination of a distinctive population^14^.

#### Mice

Single cell suspensions were prepared from spleens by mincing the tissue through a 70-μm cell strainer (BD Bioscience). Erythrocytes were lysed in a hypotonic ammonium chloride buffer. The gating strategy consists of a lymphocyte gate using the forward and side scatter signal, after which CD3^+^CD4^+^ cells were gated and subsequently CD28 expression was monitored within this gate (Supplementary Figure S1b). Surface and intracellular cytokine staining were used to identify and characterize CD4^+^CD28^null^ T cells. MOG-specific CD4^+^ T cell responses were determined after *in vitro* stimulation with MOG_35–55_ (10 µg/ml, Hooke laboratories) peptides for 8 hours (6 hours in the presence of Brefeldin A). Fluorochrome-conjugated antibodies specific for CD3, CD4, CD27, CD28, CD62L, CD127, IFN-γ and granzyme B were purchased from BD Biosciences, Biolegend, or eBioscience. Cells were acquired using a BD LSR II flow or FACSAria II cytometer, and data were analysed using FlowJo (TreeStar) or BD FACSDiva software.

### Immunohistochemistry

Mice were perfused with Ringer’s solution, spinal cords were dissected and, via a PFA/sucrose gradient, frozen in liquid nitrogen, 30 days after EAE induction. Ten micrometre cryosections were cut on the Leica CM3050S cryostat (Leica Microsystems, Wetzlar, Germany). Sections were fixed, blocked and incubated with antibodies against CD4 (1/100, BD Biosciences, 553043) and granzyme B (1/100, Abcam, Ab4059). Binding of these primary antibodies was visualized with the appropriate Alexa 488 or Alexa 555 (1/500, Life technologies, Merelbeke, Belgium) and nuclear staining was performed with DAPI (Life technologies). Autofluorescence was blocked using 0.1% Sudan Black in 70% ethanol. Demyelination and infiltration were visualized by 3, 30 diaminobenzidine (DAB) staining of myelin basic protein (MBP) with the envision kit according to the manufacturers protocol (dako Glostrup, Denmark) and subsequent hematoxylin counterstaining. In short, peroxidase activity was inhibited with 0.3% H_2_O_2_. Slides were blocked in PBS containing 10% protein block (dako Glostrup) and incubated with rat anti-mouse MBP (1/100, Millipore, MAB386) for 1 h at room temperature. Following incubation with a peroxidase labelled polymer, staining was performed with DAB substrate and hematoxylin counterstain. Microscopical analysis was performed using a multiviewer DM 2000 LED microscope and DM 4000 LED microscope with Leica Application Suite software (Leica Microsystems).

### Histological quantification

The extent of demyelination was evaluated in spinal cords of three mice per group (MCMV+ EAE, EAE control and MCMV control group). Each mouse displayed a disease score close to the median of the respective group. Every 200 µm, an entire longitudinal spinal cord section was analysed for immune infiltrates and demyelination, with a total of four sections for each animal. Demyelinated area was assessed as loss of MBP staining within the white matter of these four sections covering the entire spinal cord. Microscopical analysis was performed using a multiviewer DM 2000 LED microscope (Leica Microsystems) and Fiji software (NIH ImageJ).

### *In vitro* CMV stimulation assay

PBMCs from 12 HC and 8 MS patients were isolated from whole blood via density gradient centrifugation. These donors differed according to their CMV status and CD4^+^CD28^null^ T cell expansions (Table 2). PBMCs were cultured in RPMI-1640 medium (Lonza, Basel, Switzerland) supplemented with 10% foetal bovine serum (FBS; Hyclone Europe, Erembodegem, Belgium), 1% nonessential amino acids, 1% sodium pyruvate, 50 U/ml penicillin and 50 mg/ml streptomycin (all Life technologies).

**Table 2:** Study subjects for *in vitro* CMV stimulation assay.

	MS patients (n = 8)	Healthy controls (n = 12)
CMV+ exp+	4	4
CMV+ exp−	1	4
CMV− exp−	3	4

MS, multiple sclerosis; CMV+/−, cytomegalovirus seropositive or negative; exp+/−, CD4^+^CD28^null^ T cell expansions are present (≥2%) or not (<2%).

To mimic chronic CMV stimulation, cells were stimulated weekly with CMVpp65 recombinant protein (10 µl/ml, Miltenyi Biotec, Bergisch Gladbach, Germany) or IL-2 (5 U/ml, Roche Diagnostics, Basel, Switzerland) for a maximum of 20 days. At different time points (d0, 1, 6, 9, 12, 15 and 20), the relative number of CD4^+^CD28^null^ T cells was determined by flow cytometry as described above.

### Generation of MBP reactive T cell clones

MBP-specific T cell clones were generated as described previously^65^. Briefly, MBP-reactive T-cell lines were generated from the blood of MS patients via limiting dilution analysis (LDA), cloned with phytohemagglutinin (PHA) in the presence of allogeneic accessory cells and further expanded by successive rounds of restimulation with MBP or PHA and autologous antigen presenting cells (APCs).

### Statistical analysis

Statistical analyses were performed using GraphPad Prism version 6 and SAS 9.3. Parametric analyses include t-tests (2 groups), 1-way ANOVA and 2-way ANOVA (multiple groups). Nonparametric tests encompass Mann-Whitney tests (2 groups) and Kruskal-Wallis tests (multiple groups). Parametric data are shown as mean ± SD, nonparametric data as median ± interquartile range. A p-value < 0.05 was considered significant.

### Ethics approval and consent to participate

Experiments involving human samples and data were approved by the Medical Ethics Committee UZ KU Leuven and experiments were performed in accordance with its guidelines and regulations. Informed consents were obtained from all donors.

All animal studies were in accordance with the EU directive 2010/63/EU for animal experiments and were approved by the Ethical Committee Animal Experiments UHasselt.

## Electronic supplementary material

Dataset 1
