# Supplementary material for: Cytomegalovirus infection exacerbates autoimmune mediated neuroinflammation
Source: Sci Rep. 2017 Apr 6;7:663. doi: 10.1038/s41598-017-00645-3 (PMC5428769; doi:10.1038/s41598-017-00645-3)
Supplement: Supplementary file 1 — Dataset 1 [file 41598_2017_645_MOESM1_ESM.docx]

**Cytomegalovirus infection exacerbates autoimmune mediated neuroinflammation**

Marjan Vanheusden, Bieke Broux, Suzanne P.M. Welten, Liesbet M. Peeters, Eleni Panagioti, Bart Van Wijmeersch, Veerle Somers, Piet Stinissen, Ramon Arens, Niels Hellings


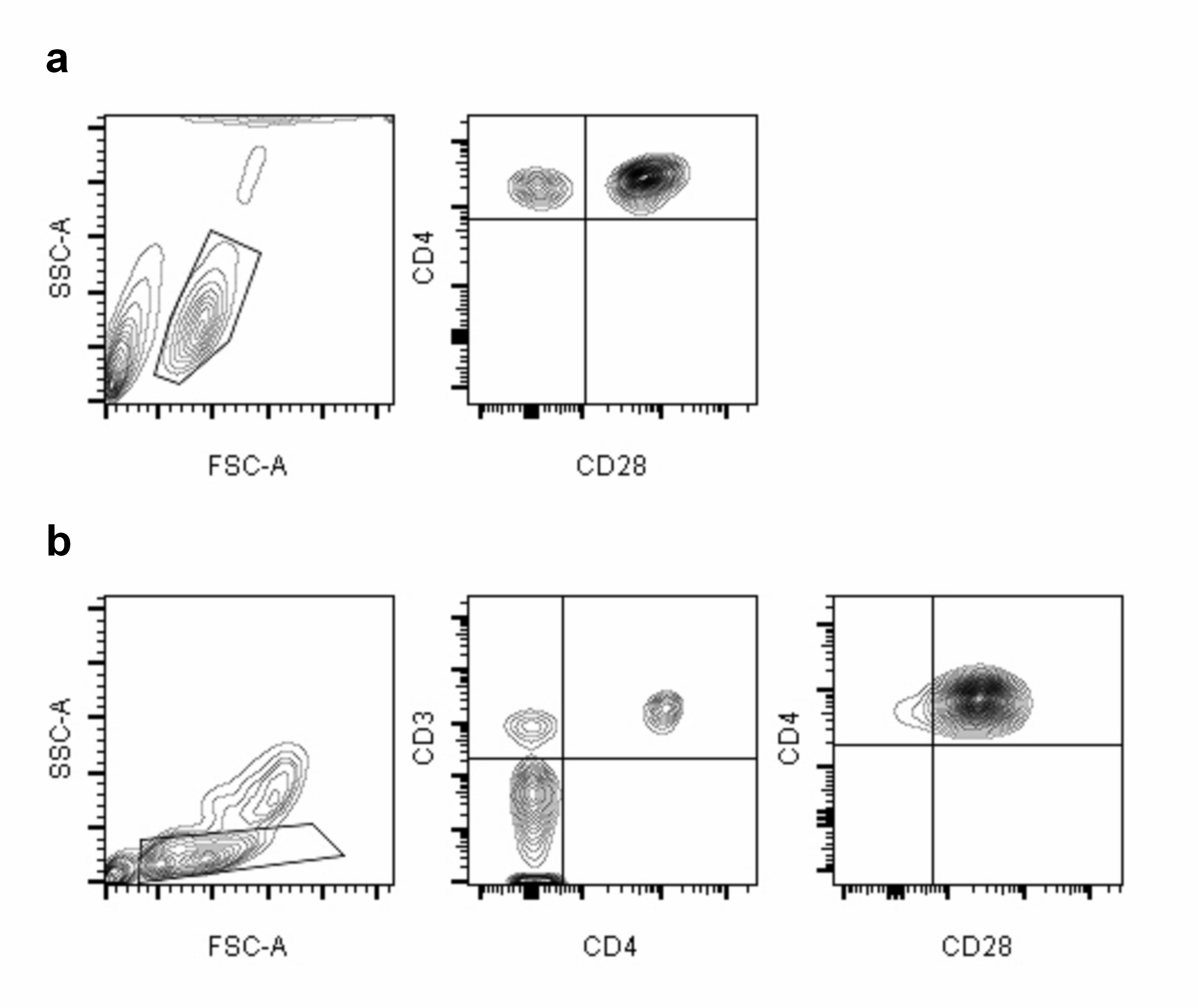


**Supplementary figure 1**: Gating strategy. (**A**) In human samples, the gating strategy consists of a lymphocyte gate using the forward and side scatter signal, after which CD4^+^ cells were gated and subsequently CD28 expression was monitored within this gate. (**B**) In mouse samples, the lymphocytes were gated, after which CD3^+^CD4^+^ cells were targeted and CD28 expression was monitored within this gate.


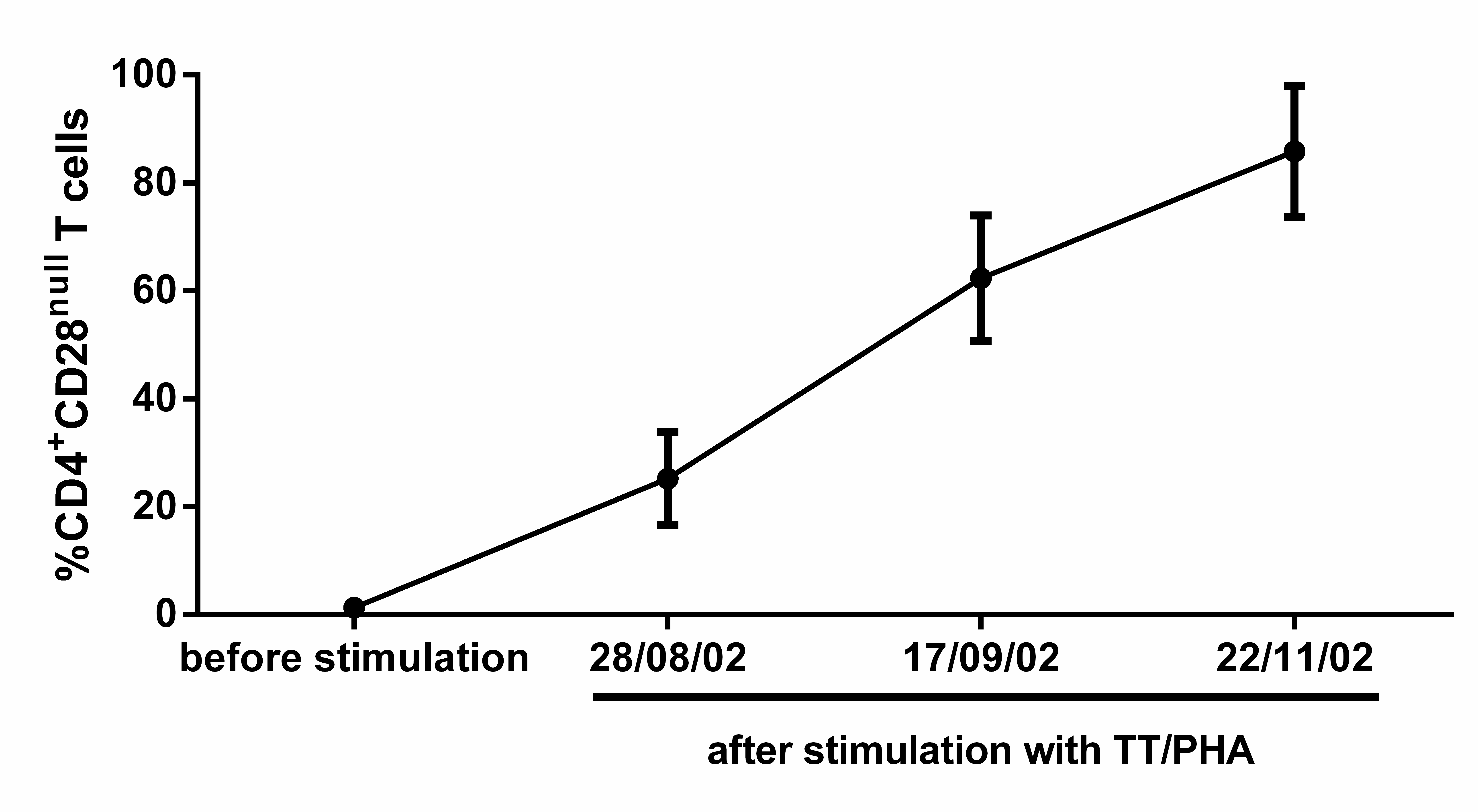


**Supplementary figure 2:** Historical human tetanus toxoid (TT) specific T cell clones repeatedly stimulated with TT/PHA (n=5) were thawed and analysed for the number of CD4^+^CD28^null^ T cells via flow cytometry.
